# The Microbiota of Homemade Tepache Includes Antibiotic-Resistant Microorganisms

**DOI:** 10.17912/micropub.biology.001744

**Published:** 2025-11-25

**Authors:** Timothy Allshouse, Myleen Amendano, Becky Caruso, Realyn Del Campo, Gwen Murphy, Lauren Shaffer, Ethan Steinberg, Aidan Sullivan, Emily Stowe

**Affiliations:** 1 Department of Biology, Bucknell University, Lewisburg, Pennsylvania, United States

## Abstract

Tepache is a traditional, homemade Mexican drink made by fermenting pineapple rinds. The natural probiotic bacteria in tepache are said to promote a healthy gut microbiome. This study assessed the microbial community in homemade tepache for diversity, survival in simulated gastric fluid, and antibiotic resistance. Simulated gastric passaging reduced total community numbers but the community density was not strongly impacted by exposure to tetracycline. Metagenomic analysis reveals a community dominated by *Bacillus, Meyerozyma* and *Talaromyces.* These results indicate that consuming home fermented beverages may provide helpful probiotic bacteria but could also expose the gut microbiome to antibiotic resistance genes.

**Figure 1. Community density and composition of homemade tepache:**
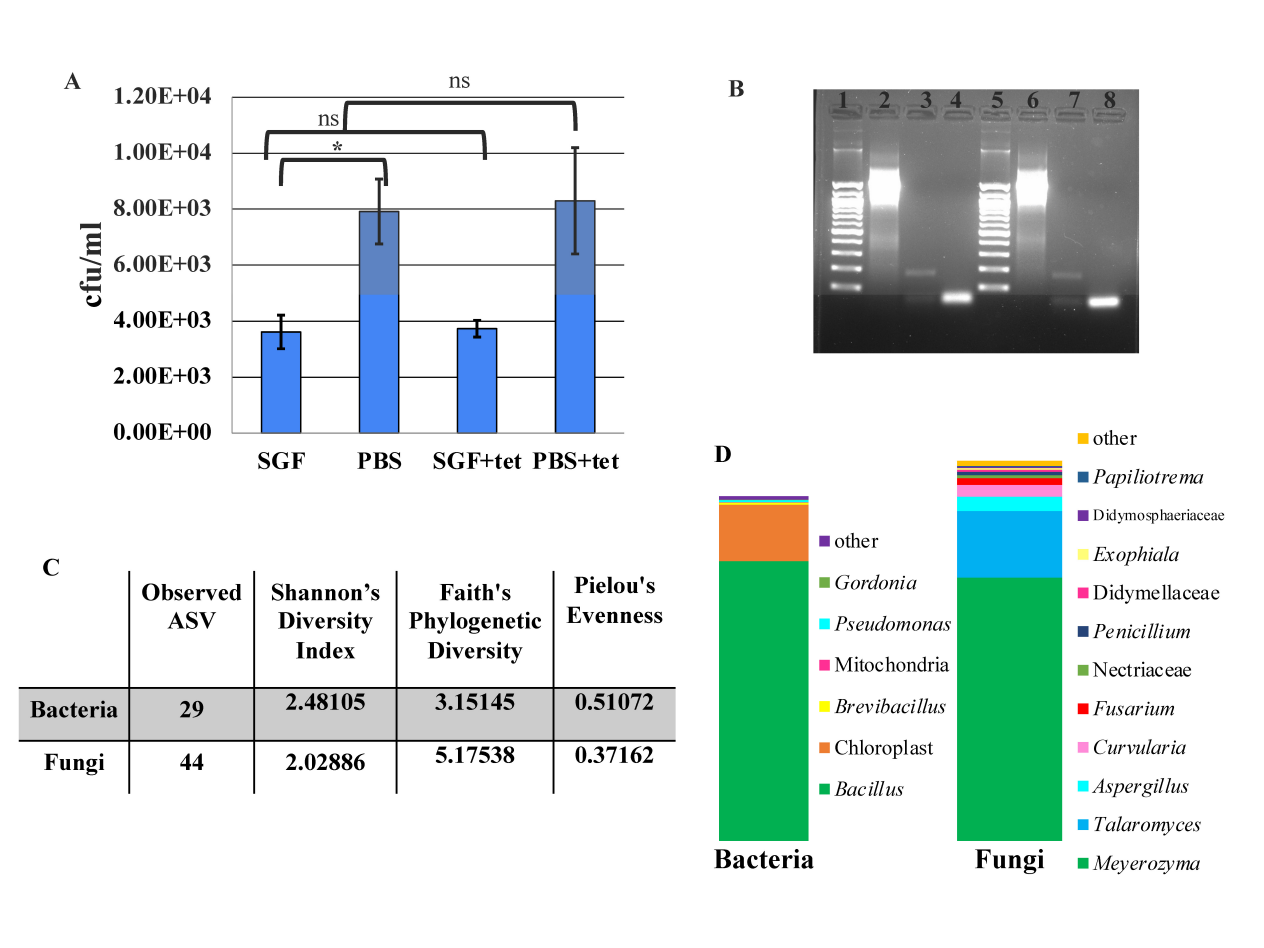
**(A)** Colony-forming units per milliliter (CFU/mL) of microbes detected in homemade tepache with (SGF: simulated gastric fluid) and without (PBS: phosphate buffered saline) gastric stress on de Man Rogosa and Sharpe (MRS) agar with and without tetracycline (tet). Paired two tailed t-test indicates a statistical difference between the control (PBS) and samples exposed to simulated gastric fluid (*, t = 12.6984, p < 0.0001) but no significant (ns) difference between control groups exposed to tetracycline, or samples exposed to simulated gastric fluid and then exposed to tetracycline. **(B)** Two independently isolated DNA samples from the same tepache were assessed for presence of antibiotic resistance genes via PCR and products were separated on a 2% agarose gel by electrophoresis. Lanes 1 and 5: Zymo 100 bp ladder, lanes 2 and 6: 16S rRNA amplicon, lanes 3 and 7 *tetM* (172 bp, observed) and *blaD* (283 bp, not observed) amplicons, and lanes 4 and 8: *aaC* (482 bp, not observed), *tetO* (515 bp, not observed) and *tetB* (634 bp, not observed). The band seen in lanes 4 and 8 are too small to be any of the expected products and likely represents primer dimer. **(C)** Alpha diversity metrics indicate a community with low diversity that is dominated by a small number of genera. ASV: Amplicon sequence variants **(D)** Community composition of tepache, most abundant genera (> 0.1%) based on amplification bacterial 16S rRNA v3/v4 region (left) and fungal ITS region (right). Other contains all ASV with <0.1% representation in the community.

## Description

Fermentation of foods and beverages increases digestibility, nutrient availability, product shelf life and reduces exposure to food borne pathogens (Soemarie et al., 2021). A diet rich in fermented foods and beverages is associated with healthy gut function, likely by supplying probiotic bacteria (O’Connor, 2021, Novianti et al., 2024). Probiotic bacteria are helpful microbes that contribute to healthy gut function (Wang et al., 2020, Gutierrez-Sarmiento et al., 2022). Probiotic beverages such as kefir and kombucha are commercially available but many consumers prefer to brew these beverages at home (Cuamatzin-Garcia et al., 2022). A less well known traditionally fermented beverage is tepache. Tepache, a traditional Mexican beverage, is produced by fermenting pineapple rinds with sugar and various spices (Aguilar-Uscanga et al., 2024). However, recent studies have indicated that some probiotic supplements have antibiotic resistance and that some of the genes for these resistance mechanisms are on mobilizable genetic elements which raises concerns regarding the transmission of antibiotic resistance genes (ARG) to the gut microbiome (Sharma et al., 2014). While commercially available probiotic supplements and foods can be routinely screened for the presence of antibiotic resistance genes (Wong et al., 2015), home fermented products cannot be similarly screened. Thus homebrewed tepache can be a good source of probiotic organisms to aid gut health maintenance, but also a potential source of ARG (Hummel et al., 2007, Anisimova et al., 2022). We tested a home-prepared batch of tepache for microbial density, microbial diversity, and acid and antibiotic resistance. We measured bacterial density with and without simulated digestion by incubating the beverage in the presence of simulated gastric fluid or PBS respectively at 37°C for 1 hour. The cell density in the control samples (PBS) on media lacking tetracycline was lower than previously reported (Fuente-Salcido et al., 2015) likely reflecting local brewing conditions. Exposure to the SGF significantly reduced total microbial numbers by approximately 55% ( Figure 1A, two tailed, paired t-test, t = 12.6984, p < 0.0001). Community density was nearly identical between control and tetracycline media in both control and gastric-stressed conditions ( Figure 1A ). We identified the presence of the *tetM* gene via PCR in community DNA, but not the other genes tested ( Figure 1B, ampicillin resistance ( *blaD* ), tetracycline resistance ( *tetO* , *tetB* ) or quinoline resistance ( *aac* )). To further explore community diversity, we sent DNA for microbiome analysis of the 16S rRNA (V3/V4 region) and the fungal ITS region. As might be expected for a fermented product, the total ASV (amplicon sequence variants) were low ( Figure 1C ) and the alpha diversity metrics indicate a community with low phylogenetic diversity (Faith’s PD), dominated by a few genera (Shannon’s Diversity Index and Pielou’s Evenness). Similar to a previous report by Gutiérrez-Sarmiento et al., (2022) the bacterial community was dominated by Firmicutes, but our community contained *Bacillus* species and not the expected lactic acid bacteria based on the results of that previous study ( Figure 1D ). A review by Cuamatzin-Garcia et al., (2022) indicates that *Bacillus* sp. have been found in traditionally brewed tepache. While *Bacillus* is less conventionally considered a probiotic genus than lactic acid bacteria, as reviewed by Romera-Luna et al (2017) many *Bacillus* species may have probiotic potential. The difference between expected community composition (Gutiérrez-Sarmiento et al., 2022) and our results highlights potential risks of homebrewed, wild fermented beverages. The chloroplast and mitochondrial sequences are identical to the 16S rRNA genes from pineapple chloroplast and mitochondria. The yeast *Meyerozyma* dominated the fungal community. Gutiérrez-Sarmiento et al. (2022) also identified this yeast genus, but our community lacked the abundant *Saccharomyces* seen in that study. These differences should be expected since tepache is produced via a wild fermentation and likely influenced by local environmental conditions. In future work we will examine the impact of production differences on the microbial community and more specifically on the level of antibiotic resistance. While the simplicity of tepache production (no requirement for a SCOBY or starter grains) makes it attractive for home production, the use of a “wild” fermentation also increases the batch to batch differences which might reduce its utility as a probiotic and also increase the potential of exposure to antibiotic resistant bacteria.

## Methods

This work was completed as part of a course-based undergraduate research experience in BIOL302 Microbiology at Bucknell University in Spring 2025. We analyzed one batch of tepache that was prepared in a home setting in February 2025. Tepache was prepared in a home setting with 3 cups of rind and core from 1 pineapple (non-organic), ½ cup of piloncillo (unrefined whole cane sugar), 4 liters of water, a cinnamon stick, and whole allspice. Once the sugar was dissolved, the pineapple and spice components were added and the mixture fermented at room temperature (~22 ℃) for four days. After fermentation, the tepache was strained and stored at 4 ℃. For probiotic bacteria to effect gut function they must survive passage through the acidic stomach. Naissinger da Silva et al. (2021) developed a protocol to assess probiotic bacteria survival after exposure to simulated aspects of digestion. Similarly, we approximated the stress of passing through the stomach by diluting tepache 1:10 with simulated gastric fluid (RICCA 7108-32, 0.2 (w/v) Sodium Chloride in 0.7% (v/v) hydrochloric acid and peptone, adjusted to a pH of 1.4.) or sterile phosphate buffered saline (PBS) and incubated at 37 °C for 1 hour with shaking. This temperature was chosen to replicate body temperature and the hour long incubation is based on gastric emptying studies that indicate an average half gastric emptying of 81 minutes for milk in children (Hauser et al., 2016). This incubation period was also easy to implement within a three hour undergraduate laboratory class. After incubation 10-fold serial dilutions in PBS were prepared and 100 μL spread on de Man Rogosa and Sharpe media (MRS) or MRS plus tetracycline (5 μg/ml). The plates were then incubated at 30 ℃ for 24 to 48 hours. CFU/mL was calculated from plates with between 30 and 300 colonies. 10-fold serial dilutions in PBS were prepared and 100 μL spread on de Man Rogosa and Sharpe media (MRS) or MRS plus tetracycline (5 μg/ml). The plates were then incubated at 30 ℃ for 24 to 48 hours. CFU/mL was calculated from plates with between 30 and 300 colonies.

Tepache community DNA was isolated using the Zymo Fecal/Soil DNA Miniprep (D6010). The isolated DNA was PCR amplified 94 °C for 4 minutes, and 35 cycles of 94 °C 30s, 55 °C (or 53 °C) for 30s, 72 °C for 90S. The amplified DNA was visualized on a 2% agarose gel using 1X TAE buffer. A positive control reaction of the 16S rRNA gene was amplified alone (FD1 AGAGTTTGATCCTGGCTCAG, RD1 AAGGAGGTGATCCAGCC, Weisburg et al, 1991) at 55 °C. Multiplex reactions amplified *tetM* (Forward: ATCCTTTCTGGGCTTCCATT Reverse: TCCGTCACATTCCAACCATA, Eitel et al., 2013) and *blaD* (Forward: AGCTTGATGCGGAATCTTACG Reverse: GCACGGTTATACGGCTGAAC, Tan et al., 2018) at 53 °C or *aac* (Forward: TTGCGATGCTCTATGAGTGGCTA Reverse: CTCGAATGCCTGGC GTGTTT, Park et al., 2006), *tetB* (Forward: CCTCAGCTTCTCAACGCGTG Reverse: GCACCTTGCTGATGACTCTT, Randall et al., 2004) and *tetO* (Forward: AACTTAGGCATTCTGGCTCAC Reverse: TCCCACTGTTCCATATCGTCA, Zhang et al., 2017) at 55 °C.

The same DNA was sent for 16S/ITS2 amplicon sequencing to SeqCenter ( https://www.seqcenter.com/ ) using 10K paired reads. Sequence analysis was conducted by Seqcenter using Qiime 2. The bacterial composition was analyzed by importing the sequences to Qiime2.1 for analysis. The primer sequences were removed using Qiime2’s cutadapt2 plugin using region V3/V4, forward trim sequence: CCTAYGGGNBGCWGCAG, and reverse trim sequence: GACTACNVGGGTMTCTAATCC. The sequences were denoised using Qiime2’s dada2 plugin, detailing each amplicon sequence variant and then using the Silva 138 99% full-length sequence database to collapse to their lowest taxonomic units. The fungal composition was analysed the same way through Qiime2.1. The primer sequences were removed using Qiime2’s cutadapt2 plugin using region ITS2, forward trim sequence: GCATCGATGAAGAACGCAG, and reverse trim sequence: TCCTCCGCTTATTGATATGC. The Unite 899% full-length sequence database was used to collapse the sequences to their lowest taxonomic units. The bacterial and fungal composition was assessed at the genus level, any ASV that were less than 0.1% of the total reads were classified as other. Sequence data can be found under bioproject accession number PRJNA1277773 and biosamples accession numbers SAMN49144880 and SAMN49144885.
